# An Online Observational Study of Patients With Olfactory and Gustory Alterations Secondary to SARS-CoV-2 Infection

**DOI:** 10.3389/fpubh.2020.00243

**Published:** 2020-05-29

**Authors:** Patricia Gómez-Iglesias, Jesús Porta-Etessam, Teresa Montalvo, Adrián Valls-Carbó, Vicente Gajate, Jordi A. Matías-Guiu, Beatriz Parejo-Carbonell, Nuria González-García, David Ezpeleta, José Miguel Láinez, Jorge Matías-Guiu

**Affiliations:** ^1^Department of Neurology, Institute of Neurosciences, IdISSC, Hospital Clínico San Carlos, Universidad Complutense de Madrid, Madrid, Spain; ^2^Department of Neurology, Hospital Quirón, Madrid, Spain; ^3^Department of Neurology, Hospital Clínico de Valencia, Universidad de Valencia, Valencia, Spain

**Keywords:** SARS-CoV-2, Covid-19, coronavirus, neurological, olfactory alterations, anosmia, online questionnaire

## Abstract

**Introduction:** Since the beginning of the Covid-19 epidemic produced by SARS2-Cov virus, olfactory alterations have been observed at a greater frequency than in other coronavirus epidemics. While olfactory alterations may be observed in patients with rhinovirus, influenza virus, or parainfluenza virus infection, they are typically explained by nasal obstruction with mucus or direct epithelial damage; in the case of SARS-CoV-2, olfactory alterations may present without nasal congestion with mucus. We performed a study of patients presenting olfactory/gustatory alterations in the context of SARS-CoV-2 infection in order to contribute to the understanding of this phenomenon.

**Material and Methods:** We performed a descriptive, cross-sectional, observational study of the clinical characteristics of olfactory/gustatory alterations using a self-administered, anonymous online questionnaire.

**Results:** A total of 909 patients with SARS-CoV-2 infection and olfactory/gustatory alterations responded to the questionnaire in the 4-day data collection period; 824 cases (90.65%) reported simultaneous olfactory and gustatory involvement. Patients' responses to the questionnaire revealed ageusia (581, 64.1% of respondents), hypogeusia (256, 28.2%), dysgeusia (22, 2.4%), anosmia (752 82.8%), hyposmia (142, 15.6%), and dysosmia (8, 0.9%). Fifty-four percent (489) did not report concomitant nasal congestion or mucus.

**Conclusion:** Olfactory alterations are frequent in patients with SARS-CoV-2 infection and is only associated with nasal congestion in half of the cases.

## Introduction

Prior to December 2019, 6 coronaviruses (CoV) had been reported to infect humans: 2 αCoV (HCoV-229E and HCoV-NL63) and 4 βCoV (HCoV-OC43, HCoV-HKU1, SARS-CoV, and MERS-CoV). The latter 2 viruses have caused epidemics in Asia and the Middle East. In 2003, SARS-CoV was identified as the cause of severe respiratory symptoms that had appeared for the first time in 2002 in Guangdong province, China; patients presented fever, myalgia, dyspnoea, and lymphocytopaenia, which could lead to pneumonia and death. The virus was spread through the air and close contact with infected individuals. On 31 December 2019, the World Health Organization reported the detection of a novel CoV (SARS-CoV-2) in patients with pneumonia in the city of Wuhan, in the Chinese province of Hubei, subsequently spreading rapidly through China and the rest of the world. The novel virus is classified as a βCoV, and although it displays striking similarities to SARS-CoV, it is genetically and structurally different (1, 2) and generates the disease that has been named Covid-19. One important characteristic of the new virus is its high transmission rate (3); it is more contagious than SARS-CoV (4), spreading through respiratory droplets and close contact with patients and infected objects; it can also be spread by asymptomatic infected individuals (5–7). The epidemic reached Spain in February 2020, with over 80 000 confirmed cases currently recorded.

Although various observational studies have analyzed the symptoms of the disease, few studies have reported neurological symptoms, with the exception of headache and vestibular symptoms in observational studies (8–13), a specific study of these symptoms in hospitalized patients (14), and isolated case reports (15). However, some authors suggest that the virus may affect the central nervous system (CNS), similarly to SARS-CoV, which was detected in the brains of some patients (16). The ongoing clinical experience with patients with SARS-CoV-2 infection has revealed a high frequency of olfactory alterations, both in Spain and in other countries, although few data have been published on the subject. While olfactory alterations may be observed in patients with rhinovirus, influenza virus, or parainfluenza virus infection, they are typically explained by nasal obstruction with mucus or direct epithelial damage; in the case of SARS-CoV-2, olfactory alterations present without nasal congestion with mucus. We performed a study of patients presenting olfactory/gustatory alterations in the context of SARS-CoV-2 infection in order to contribute to the understanding of this phenomenon.

## Materials and Methods

We performed a descriptive, cross-sectional, observational study of the clinical characteristics of olfactory/gustatory alterations using a self-administered, anonymous online questionnaire with some open-ended questions (Supplementary Material 1), targeted at the population meeting the eligibility criteria mentioned below and freely volunteering to participate in the study. The questionnaire was accessible through a Google document (docs.google.com) and publicized on social media with the assistance of the Spanish Society of Neurology. Patients implicitly agreed to participate by completing the questionnaire, which was available on an online platform accessible to the target population and the study met the ethical requirements. Individuals who either had a medical diagnosis of SARS-CoV-2 infection (positive test results), or who were under quarantine due to compatible viral symptoms pending testing, were asked to complete the questionnaire. Effectively, patients who participated in the study had been diagnosed by the doctors responsible for care in the pandemic for presenting symptoms such as dry cough, dyspnea, fever, among others, and being in a contagious environment, for which reason they had been indicated at least fifteen days of quarantine, following the instructions of the health administration, which at that time did not require the conformity of serological studies for the diagnosis. Furthermore, all the included patients presented alterations in taste or smell. Data were recorded in a database between 20 and 24 March 2019. The day before the closure of the data collection period (23 March), official sources reported a total of 35 212 confirmed cases in Spain (Spanish Ministry of Health) and the number of 80,000 cases was exceeded by March 31.

The questionnaire comprised 4 sections: (1) Demographic data, including age, sex, medical history, and risk factors; (2) Characteristics of olfactory alterations, defined as anosmia (complete absence of olfaction), hyposmia (reduced olfaction, with at least 2 types of smell preserved), dysosmia (reduced olfaction with presence of unpleasant smells), and other (including difficult to define sensations) and gustatory alterations, defined as defined as ageusia (complete absence of tast), hypogeusia (reduced taste,), dysgeusia (reduced and unpleasant taste), and other (3) Temporal pattern of onset of olfactory alterations (concurrently with other symptoms of infection; after onset of viral infection symptoms; no other symptoms; and other [not classifiable]); (4) Temporal pattern of resolution of olfactory alterations (before resolution of other symptoms; concurrently with resolution of viral infection symptoms; persisted in isolation after resolution of other symptoms; persisted [no other symptoms presented]; and other [not classifiable]); (5) Time from onset to resolution of olfactory alterations, if applicable. The questionnaire included similar questions about gustatory alterations. Despite the difficulty of designing an online questionnaire to assess changes in taste and smell, this study was also specially designed to define patient profiles and chronology.

The data recorded include both patients testing positive for SARS-CoV-2 and those under quarantine due to compatible symptoms. Statistical analysis of data was conducted using the SPSS statistics software package. Data are expressed as percentages.

## Results

A total of 909 patients completed the questionnaire. At the time they completed the questionnaire, 67 (7.4%) had been tested for the virus and 842 (92.6%) had not. A total of 626 (68.9%) respondents were women and 283 (31.1%) were men; mean age was 34 (range, 16–74). All patients reported gustatory or olfactory alterations, with 845 (92.95%) reporting gustatory and 888 (97.7%) reporting olfactory alterations. Simultaneous involvement of both senses was reported in 824 cases (90.65%). Gustatory alterations were classified as ageusia (64.1% of respondents), hypogeusia (28.2%), and dysgeusia (2.4%). Olfactory alterations were classified as anosmia (82.8%), hyposmia (15.6%), and dysosmia (0.9%). Fifty-four percent of the 906 respondents who answered the question (n = 489) did not present concurrent nasal congestion/mucus. The most frequent associated symptoms were myalgia (296 patients; 32.5%), dry cough (199; 21.9%), fever (170; 18.7%), and other symptoms (dyspnoea, headache, odinophagia, gastrointestinal alterations, nasal congestion [19; 2.1%]). Olfactory/gustatory alterations took slightly longer than 6 days to resolve. The associated factors are listed in Supplementary Material 2; no statistically significant associations were identified. These data are summarized in Tables 1, 2.

**Table 1:** Presence of gustatory and olfactory alterations in patients testing positive for SARS-CoV-2 or under quarantine for compatible symptoms.

**Gustatory**			**Olfactory**		
	**Quarantine**	**Positive test result**		**Quarantine**	**Positive test result**
Ageusia	49.3%	61.4%	Anosmia	81.7%	76.1%
Hypogeusia	37.3%	25.8%	Hyposmia	15.1%	17.9%
Dysgeusia	10.5%	2.0%	Dysosmia	0.7%	3.0%
Other	0.9%	10.8%	Other	0.6%	3.0%

**Table 2:** Temporal profile of olfactory/gustatory alterations.

Onset of symptoms (gustatory/olfactory)^*^	
Simultaneous with other symptoms	26%
After onset of other symptoms	23.9%
No other symptoms	38.2%
Other	11.9%
**Symptom resolution**	
Before resolution of other symptoms	3.3%
Simultaneous with other symptoms	5.2%
Persists in isolation after resolution of other symptoms	42.9%
Persists (no other symptoms presented)	31.3%
Other	17.3%
Time from onset to resolution of gustatory/olfactory symptoms	6.3 days

* *Gustatory and olfactory alterations were analyzed in combination, not individually*.

## Discussion

Our findings show that olfactory alterations are more frequent in patients with SARS-CoV-2 infection than in other viral infections, although the incidence of this symptom is yet to be determined. The sample analyzed corresponds to a young population (mean age of 34.7 years), although this may reflect a selection bias, as the younger population is more familiar with the use of the Internet and social media. No differences were observed between patients who had been tested for SARS-CoV-2 infection and those who were under quarantine. Olfactory alterations could suggest the possibility of the virus entering the CNS as the result of the frequency of nasal congestion. Onset of these symptoms occurs early in the course of the disease, presenting simultaneously with other symptoms in more than half of cases. These sensory deficits are severe: a high percentage of patients reported complete loss of the sense of smell/taste (anosmia/ageusia), which negatively impacts on quality of life. In the majority of cases, olfactory/gustatory alterations were the only manifestation or were accompanied by mild viral symptoms; only 2.1% of patients reported more severe symptoms, such as dyspnoea. Compared against patients who had been tested for SARS-CoV-2, patients under quarantine more frequently presented olfactory/gustatory alterations in isolation (244; 25.6%); this may partly explain the high percentage of patients who had not been tested.

It is yet to be determined whether SARS-CoV-2 reaches the CNS, like other viruses. Viruses can enter the CNS by the retrograde neuronal or the haematogenous (17, 18) routes. In the former, a virus infects peripheral neurons and accesses the CNS via their axons (19–21); it may enter in the brain via the olfactory nerve olfactory bulb, or through the cribiforme plate (22, 23), through the trigeminal nerve, or through sensory fibers of the vagus nerve (24–27). Therefore, olfactory alterations may indicate spread of the virus to the CNS (28) and the possibility of delayed neurological symptoms. Regarding this point, a recent study drew attention to the high risk of CNS infection due to the greater local expression of ACE2 receptors, highlighting the need for greater understanding of the mechanisms by which the virus interacts with the host's CNS (29).

Our study is the first to analyse the presence of olfactory alterations secondary to SARS-CoV-2 infection; the incidence of this manifestation is probably high, and is only associated with nasal congestion in half of the cases. However, we should emphasize the limitation that the study does not address the frequency of this symptom in the total patient population, as well as the limitations inherent to the methodology used. Furthermore, access to the questionnaire was conditional upon factors related to respondents' access to the Internet and social media, and hospitalized patients with more severe symptoms would not have been able to complete it. Therefore, our sample is probably composed of patients with less severe symptoms. The low number of confirmatory tests and the low possibility that the olfactory alteration was caused by another intercurrent infection are also limitations. Online questionnaires have been used in specific circumstances in which accessing patients represents a challenge (30); the restrictions associated with the situation of the epidemic favor the use of this method, although the risk of selection bias is inevitable.

## Data Availability Statement

The raw data supporting the conclusions of this article will be made available by the authors, without undue reservation.

## Ethics Statement

Ethical review and approval was not required for the study on human participants in accordance with the local legislation and institutional requirements. Written informed consent for participation was not required for this study in accordance with the national legislation and the institutional requirements.

## Author's Note

During the editorial process of the article, articles have been published in early version, and which are of interest.

*Reviews*: Wee LE, Chan YFZ, Teo NWY, Cherng BPZ, Thien SY, Wong HM et al. Eur Arch Otorhinolaryngol. 2020 doi: 10.1007/s00405-020-05999-5 (online April 24). This article supports the use of self-surveys for evaluation of olfactory disorders, in Covid 19; Xydakis MS, Dehgani-Mobaraki P, Holbrook EH, Geisthoff UW, Bauer C, Hautefort C Lancet Infect Dis. 2020 doi: 10.1016/S1473-3099(20)30293-0 (online April 15). It is an editorial comment in line with our article in which it is noted that is noteworthy that the olfactory alterations of Covid 19 do not correlated with alterations such as rhinorrhea similar to our considerations.; Lovato A, by Filippis C. Ear Nose Throat J. 2020. doi: 10.1177/0145561320920762 (online April 13). It is a meta-analysis of 5 studies. Interesting, phharyngodynia was only present in 12.4% of patients, nasal congestion in 3.7%, and rhinorrhea was rare;. Dufowt R and Rusell K (online April 13), ACS Chem Neurosci, doi: 10.1021/ascchemneuro.0c00172.

*Research articles*: Yan CH, Faraji F, Prajapati DP, Ostrander BT, DeConde AS. Eur Arch Otorhinolaryngol. 2020. doi: 10.1007/s00405-020-05999-5 (online April 24), is a survey similar to our article; Beltrán-Corbellini Á, Chico-García JL, Martínez-Poles J, Rodríguez-Jorge F, Natera-Villalba E, Gómez-Corral J, et al. Eur J Neurol. 2020. doi: 10.1111/Jan.14273 (online April 22). Case and control of 40 covid and influenza patients. Moein ST, Hashemian SMR, Mansourafshar B, Khorram-Tousi A, Tabarsi P, Doty RL. Int Forum Allergy Rhinol. 2020 doi: 10.1002/alr.22587; Yan CH, Faraji F, Prajapati DP, Boone CE, DeConde AS. Int Forum Allergy Rhinol. 2020. doi: 10.1002/alr.22579 Int Forum Allergy Rhinol. 2020. doi: 10.1002/alr.22579 (online April 17) Smell and taste loss were reported in 68% (40/59) and 71% (42/59) of Covid-19-positive subjects. Smell and taste impairment were independently and strongly associated with Covid-19-positivity; Hopkins C, Surda P, Kumar N. Rhinology. 2020 Apr 11. doi: 10.4193/Rhin20.116 (online April 11th) A similar survey, with similar limitations in relation to the respondents; Eliezer M, Hautefort C, Hamel AL, Verillaud B, Herman P, Houdart E, Eloit C. JAMA Otolaryngol Head Neck Surg. 2020 Apr 8. doi: 10.1001/jamaoto.2020.0832 (online April 8). Authors reported a case of association; Lechien JR, Chiesa-Estomba CM, De Siati DR, Horoi M, Le Bon SD, et al. Eur Arch Otorhinolaryngol. 2020. doi: 10.1007/s00405-020-05965-1 (online April 6). It is the most powerful study also confirms that patients with patients without nasal involvement are associated with olfactory disorders.

## Author Contributions

Principal investigator: JP-E. Study design and drafting of manuscript: PG-I and JP-E. Preparation of questionnaires: PG-I, JP-E, and BP-C. Creation of web page and tables: PG-I. Coordination: JP-E, DE, JL, and NG-G. Database: PG-I, TM, AV-C, and VG. Data analysis: PG-I, JP-E, and JAM-G. Analysis of results: PG-I, JP-E, NG-G, JAM-G, and JM-G. Revision of manuscript: all authors.

## Conflict of Interest

The authors declare that the research was conducted in the absence of any commercial or financial relationships that could be construed as a potential conflict of interest.
