# Supplementary material for: An Online Observational Study of Patients With Olfactory and Gustory Alterations Secondary to SARS-CoV-2 Infection
Source: Front Public Health. 2020 May 29;8:243. doi: 10.3389/fpubh.2020.00243 (PMC7273853; doi:10.3389/fpubh.2020.00243)
Supplement: Supplementary file 1 [file Data_Sheet_1.PDF]

Supplementary Material 1

The online questionnaire used in the study (English translation of the Spanish-language questionnaire).

Questionnaire about olfactory/gustatory symptoms associated with COVID-19 infection<br>- Sex<br>- Age<br>- Do you have high blood pressure?<br>- Do you have diabetes?<br>- Do you smoke?<br>- Do you use drugs?<br>- Do you have seasonal allergies (hayfever, dust mites, spring time, animals)?<br>- Have you had any severe head trauma? If so, at what age?<br>- Do you have any ear, nose, or throat disease?<br>- Do you work with organic solvents?<br>- Do you have any autoimmune disease (eg, Sjögren syndrome)?<br>- Have you taken any of the following drugs? Amlodipine, atenolol, bisoprolol, metoprolol, carvedilol, cadmium, ciprofloxacin, cocaine, diltiazem, doxycycline, enalapril, lovastatin, methotrexate, nifedipine, silver nitrate, none, other<br>- Have you taken any of the following drugs? Acetazolamide, allopurinol, captopril, dipyridamole, enalapril, flurazepam, hydrocortisone, levodopa, lisinopril, lithium, losartan, nitroglycerin, spirolactones, none, other<br>- Have you experienced any symptoms affecting your sense of taste? Yes/no<br>- If so, select the statement that best describes your symptoms: I cannot identify any taste/I can only identify some tastes/the tastes of some foods have changed/food tastes unpleasant/other<br>- Have you experienced nasal congestion or mucus at the same time as the taste/smell alterations?<br>- Have you experienced any symptoms affecting your sense of smell? Yes/no<br>- If so, select the statement that best describes your symptoms: I cannot identify any smell/I can only identify some smells/things smell differently to the way I remember/other<br>- Have you experienced taste or smell alterations in the past? Yes/no<br>- When did the taste/smell alterations start? Before the COVID-19 symptoms/at the same time as the COVID-19 symptoms/after the COVID-19 symptoms had resolved/I have not had any other symptom/other<br>- If the taste/smell alterations have resolved, how long did they last? They persisted after the other symptoms/they persisted even though I have had no other symptoms/they had the same duration as the other symptoms/they had a shorter duration than the other symptoms/other<br>- How many days passed between onset and resolution of the taste/smell alterations? Answer only if these symptoms have resolved; open-ended question.<br>- How did the taste/smell alterations first appear? Only my sense of taste was affected and the symptoms appeared suddenly/only my sense of smell was affected and the symptoms appeared suddenly/both senses were affected and

the symptoms appeared suddenly/only my sense of taste was affected and the symptoms appeared gradually (hours, days)/only my sense of smell was affected and the symptoms appeared gradually (hours, days)/both senses were affected and the symptoms appeared gradually (hours, days)/other

- Which of the following symptoms of the viral infection have you experienced? Fever, dry cough, myalgia (muscle pain), gastrointestinal symptoms (vomiting, diarrhoea, nausea), none, other
- Have you been tested for COVID-19?
- If so, what was the result? Positive/negative
