# Supplementary material for: An Online Observational Study of Patients With Olfactory and Gustory Alterations Secondary to SARS-CoV-2 Infection
Source: Front Public Health. 2020 May 29;8:243. doi: 10.3389/fpubh.2020.00243 (PMC7273853; doi:10.3389/fpubh.2020.00243)
Supplement: Supplementary file 2 [file Data_Sheet_2.PDF]

Supplementary material 2

Demographic and clinical characteristics of our sample of patients testing positive for the virus or under quarantine due to compatible symptoms.

	Positive test result	Quarantine
Mean age (years)	36.6	34.7
Sex	68.9% women, 31.1% men	68.7% women, 31.3% men
AHT	0%	3.3%
DM	0%	0.7%
Smokers	7.5%	17.6%
Drug use	3.0%	3.4%
Allergies	34.3%	34.3%
Head trauma	3.0%	1.0%
Nasal/sinus conditions	9%	5.3%
Organic solvents	1.5%	1.5%
Chemotherapy/head and neck radiotherapy	0%	1.3%
Autoimmune diseases	13.4%	3.2%
No pharmacological treatment	59.7%	51.2%

AHT: arterial hypertension; DM: diabetes mellitus.
